# Optimising the transport properties and reactivity of microbially-synthesised magnetite for *in situ* remediation

**DOI:** 10.1038/s41598-018-21733-y

**Published:** 2018-03-09

**Authors:** Nimisha Joshi, Feixue Liu, Mathew Paul Watts, Heather Williams, Victoria S. Coker, Doris Schmid, Thilo Hofmann, Jonathan R. Lloyd

**Affiliations:** 1 Williamson Research Centre for Molecular Environmental Science, School of Earth and Environmental Science, University of Manchester, Manchester, M13 9PL UK; 2 College of Life Sciences, University of Dundee, Dundee, DD1 5EH Scotland UK; 3 School of Earth Sciences, The University of Melbourne, Parkville, VIC Australia; 4 Nuclear Medicine Centre, Central Manchester University Hospitals, Oxford Road, Manchester, M13 9WL UK; 5 Department for Environmental Geosciences and Environmental Science Research Network, University of Vienna, Althnastrasse 14, 1090 Vienna, Austria

## Abstract

Engineered nanoparticles offer the potential for remediation of land and water that has been contaminated by organics and metals. Microbially synthesized nano-scale magnetite, prepared from Fe(III) oxides by subsurface Fe(III)-reducing bacteria, offers a scalable biosynthesis route to such a nano-scale remediation reagent. To underpin delivery of “bionanomagnetite” (BNM) nanomaterial during *in situ* treatment options, we conducted a range of batch and column experiments to assess and optimise the transport and reactivity of the particles in porous media. Collectively these experiments, which include state of the art gamma imaging of the transport of ^99m^ Tc-labelled BNM in columns, showed that non-toxic, low cost coatings such as guar gum and salts of humic acid can be used to enhance the mobility of the nanomaterial, while maintaining reactivity against target contaminants. Furthermore, BNM reactivity can be enhanced by the addition of surface coatings of nano-Pd, extending the operational lifetime of the BNM, in the presence of a simple electron donor such as hydrogen or formate.

## Introduction

Iron based nanoparticles including composite particles can be used to treat a variety of toxic organic compounds and heavy metals and show significant potential for industrial and remedial applications^1^. Amongst the nanomaterials that are available for use, chemically synthesized nano scale zero valent iron (nZVI) and magnetite have been most commonly used for *in situ* remediation techniques, as these can treat (via sorption and redox reactions) both, heavy metals and persistent organic compounds^2–4^. For example, magnetite and ZVI iron nanoparticles have been shown to be effective for the removal of toxic heavy metals including Cr(VI), Ni(II), Hg(II), Cd(II) and Pb(II)^5–7^ and can also promote the reductive dehalogenation of organic compounds such as trichloroethane (TCE) and perchloroethylene (PCE)^8^. Synthesis of such nanoparticles is achieved largely by chemical processes that are fast, inexpensive and provide well defined size distributions of the end product. However, these processes often involve the use of relatively extreme conditions, including high temperature regimes and harsh chemicals.

Microorganisms can offer an alternative green route to synthesize functional nanoparticles, which is scalable and cost effective, producing materials with high sorption characteristics and catalytic properties, and able to remediate a variety of target contaminants. Bionanomagnetite (BNM) is one such nanomaterial that is synthesized by Fe(III)-respiring subsurface bacteria e.g. *Geobacter sulfurreducens* and *Shewanella oneidensis*^9^, in the presence of an electron donor such as lactate, acetate or hydrogen and an insoluble Fe(III) electron acceptor, including waste iron materials^10^.

The resultant nanomaterial is highly reactive against redox active pollutants due to an abundance of Fe(II) on the surface of material and within the magnetite structure^11^. Its synthesis can be regarded as a green chemistry process, operating at ambient pressures and temperatures in the absence of toxic reagents and capping agents, and it is also amenable to surface engineering for improved reactivity. For instance, BNM has been shown to abiotically reduce Pd(II) to Pd(0), producing a nanoscale heterostructure with extended reactivity against inorganic^12,13^ and organic substrates^14^ in the presence of external electron donors. Recent studies have shown that BNM synthesis is both tunable (with respect to particle size and magnetic properties) and scalable underpinning future commercial exploitation^10,15^. Further recent studies on the potential applications of BNM have shown that it can reduce model Cr(VI) solutions both in batch reactors^16^ and in column systems^13^. Furthermore, it has been effective in reducing and source stabilization of Cr(VI) in leachates procured from chromite ore processing residue (COPR), from a contaminated landsite in Glasgow^12^.

Targeted *in situ* applications of nanoparticles for contaminant land treatment will rely on delivery of the materials via direct injection techniques, where a highly concentrated nanomaterial slurry is injected at high flow velocities into the aquifer^17^. This subsurface injection primarily aims to remediate the contaminant through reduction (transformation) and immobilization^18^ and relies on transport of the reactants from the injection point to the zone that requires remediation. A second approach involves the generation of a permeable reactive barrier, a matrix of immobile nanoparticles that have been placed in the subsurface, and aimed at treating a plume of contaminated groundwater as it passes through the barrier^3,19^. In this second scenario, mobility of the nanomaterial is not desirable.

For *in situ* applications, properties of nanomaterials are optimized to regulate deposition kinetics and aggregation behaviour. Addition of stabilizers^20^, e.g. polyelectrolytes^21^, polysaccharides^22,23^, amino acids^24^ or organic matter^25–28^, has been shown to improve the dispersal of nanomaterials by either electrostatic or steric stabilization mechanisms, thus enhancing their transport properties. The hydrochemical and hydrogeological characteristics of aquifers, such as the pH and ionic strength of the groundwater^29–32^, and the properties of the aquifer materials themselves, also influence the transport and reactivity of any nanoparticles delivered into the subsurface for remediation applications.

In this study we investigate for the first time both, the transport behaviour of biogenic nanomagnetite (BNM) and its reactivity. Transport studies used columns filled with a porous medium, representative of subsurface aquifer materials, and further demonstrate that with the use of non-toxic and inexpensive stabilizers e.g. guar gum and humic acid salts, the mobility of BNM can be controlled without affecting its remediation and sorbent properties significantly. We have used Cr(VI) as a model redox active contaminant in batch reactors, as it is a potent carcinogen that is released from mining and other industrial activities, and has a maximum permissible concentration of 50 ppb in potable water, as recommended by the World Health Organization (WHO). Batch Cr(VI) reduction studies were conducted alongside column transport studies, to investigate the possible impact of stabilizers on reactivity. Furthermore, a novel 2D-gamma imaging method was used to investigate the transport of coated and uncoated BNM in columns. To do this, BNM was labelled with the γ-emitting metastable isomer of ^99m^Tc, offering an efficient, non-invasive technique to investigate the transport and deposition behaviour of BNM in porous media. The implications for *in situ* treatment of a range of contaminants by BNM are discussed.

## Results

### Characterization of stabilized bionanomagnetite

**Figure 1:**
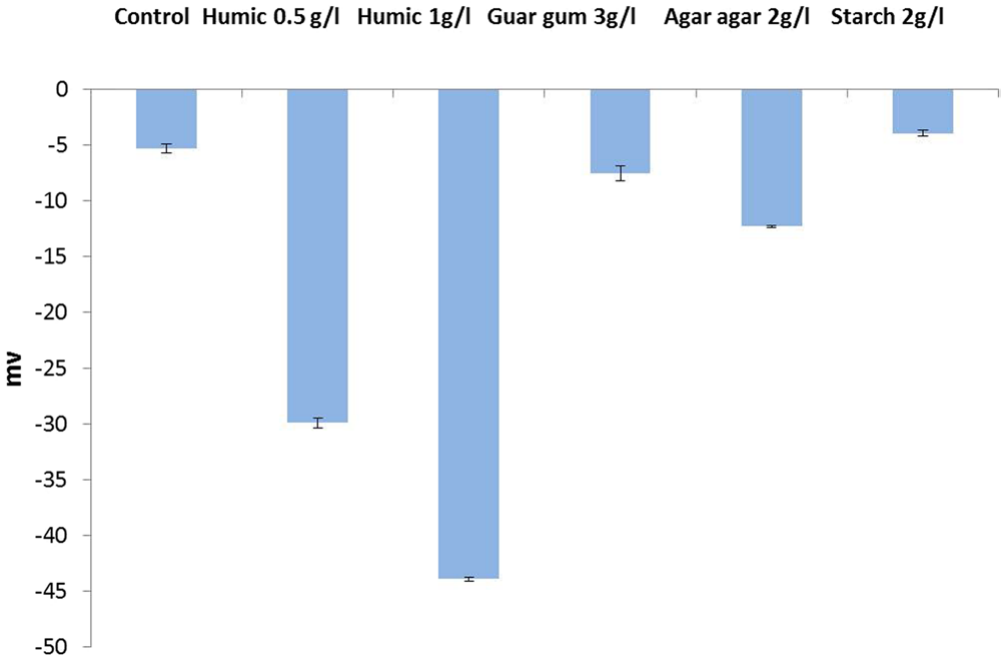
Zeta potential values for bare and stabilized BNM containing starch, guar gum, agar agar and sodium salt of humic.

**Figure 2:**
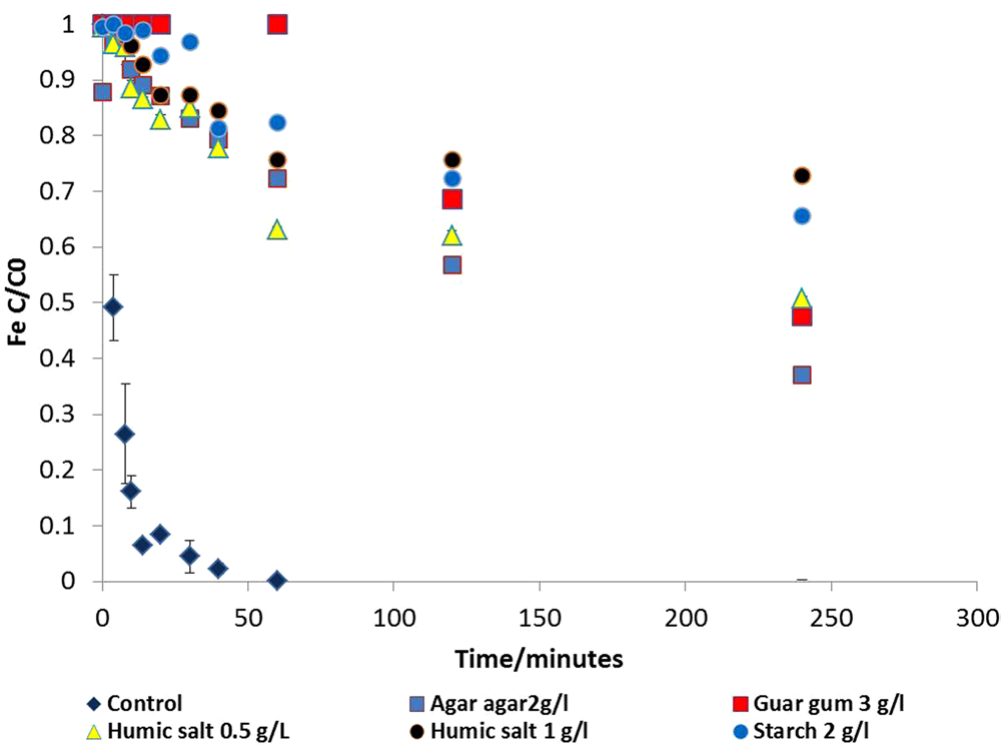
Sedimentation analysis for stabilized BNM, total iron concentration measured at each time point, C normalized to the total Fe present in the slurry (C0).

BNM was synthesized using washed cell suspensions of *Geobacter sulfurreducens*, added to a slurry containing ferrihydrite as the terminal electron acceptor and sodium acetate as the electron donor. After 48 hours of incubation, the ferrihydrite was transformed to nano-scale bionanomagnetite (BNM). An X-ray diffraction (XRD) analysis of the material indicates that the transformation was complete and that magnetite was present with minor siderite (supplementary information, Fig. S3), and X-ray magnetic circular dichroism (XMCD) data (Fig. S4a) derived from X-ray absorption spectra (XAS) within a magnetic field confirmed the presence of magnetite with an Fe(II):Fe(III) ratio of 0.58:1 in agreement with previous measurements for biogenic magnetite^33,34^. Previous Transmission electron microscopy (TEM) studies have shown that primary particles of BNM have a mean diameter of approximately 13 nm^35^, which agrees with the images of BNM particles (Fig. S4b). In this current work we noted that the primary particles were prone to agglomeration and formed aggregated particles with an average diameter of 3.5 µm measured using a time-of-transition based particle analyser (Eyetech) and a surface area of 17.92 m^2^/g. A range of surface coatings were added to the BNM to control the mobility and reactivity, and the coated materials were analysed for key parameters including size, zeta potential, viscosity and mobility in column studies. The addition of the coatings did not reduce the size of the BNM aggregates (with the exception of the humic coating), presumably due to aggregation of the individual particles that could have occurred prior to addition of coatings. For example, at pH 7.2, the d50 (median diameter of particles) for the uncoated BNM and those coated with agar agar (volume based size estimation) was 5.7 and 5.5 µm, although the individual crystallites for uncoated BNM would have been in the order of 10–50 nm^35^. The addition of guar gum and starch increased the d50 size to 12.3 and 22.4 µm respectively. In contrast, the addition of a humic acid salt coating led to a reduction in the measured d50 value to 2.67 µm (Supplementary Table S1). Addition of starch, agar agar and guar gum coatings had little impact on the zeta potential of the BNM suspensions, although a significant change was noted when a humic acid salt coating was applied to the BNM (Fig. 1). At near neutral pH, all the coated nanomaterials showed an overall negative charge.The viscosity of the coating reagents was measured in synthetic soft water at the equivalent concentrations used for subsequent mobility studies. This lead to an increase in viscosity to 2.83 × 10^−3^ and 7.94 × 10^−3^ kg/ms respectively for agar agar and guar gum, compared to 1.71 × 10^−3^ kg/ms for the background soft water matrix that was used for suspending the BNM slurry. Sedimentation studies showed that the addition of the coatings increased the stability of the suspensions in the soft water (Fig. 2). The uncoated BNM precipitated within an hour, whereas the coatings maintained 30–80% of particles in suspension for the duration of the four-hour experiment. The starch and humic coatings remained suspended for over 24 hours (data not shown). The stability of the suspensions was in the following order (final concentration of the stabilizer in brackets); humic stabilized BNM (1 g/l) > starch (2 g/l) > humic (0.5) g/l > guar (3 g/l) > agar agar(2 g/l) > control.

### Impact of stabilizers on mobility of bionanomagnetite

**Figure 3:**
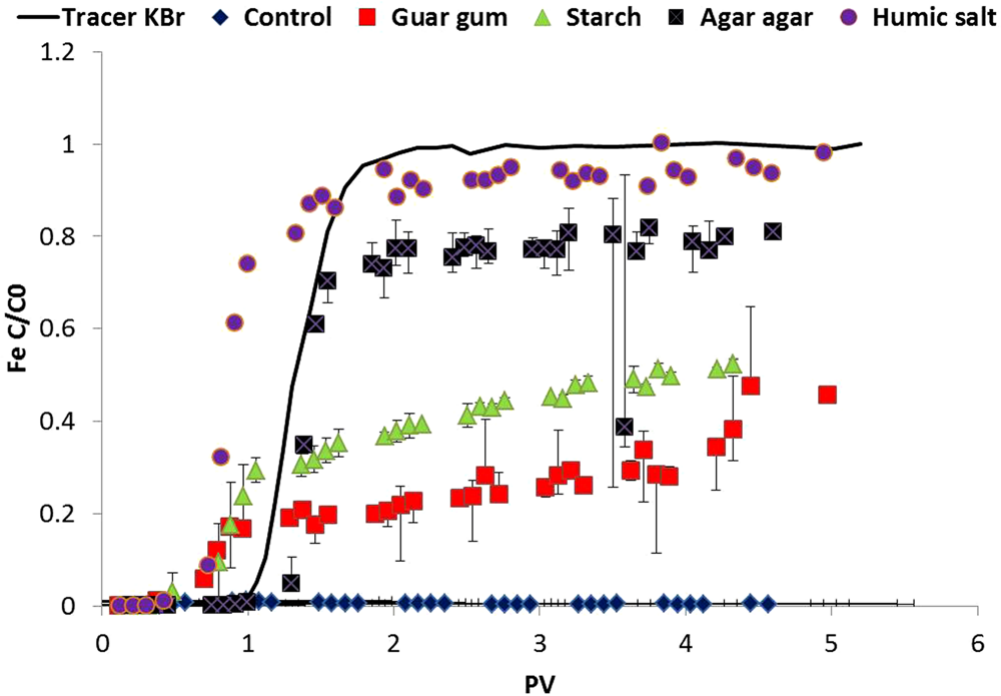
Experimental breakthrough curves for transport studies of uncoated and stabilized BNM at an injection velocity of 100 m/d. The concentration of BNM collected at each time point C normalized to total concentration plotted against pore volume (PV).

The aggregated BNM, present in slurries of both uncoated and stabilized nanomaterials (at a loading of 1 g/l total iron in synthetic soft water) were injected into columns packed with quartz sand, in a matrix of soft water at a velocity of 100 m/d representative of the injection velocities employed during *in situ* remediation applications. The uncoated BNM showed negligible mobility over the experimental period, with a maximum breakthrough (total Fe C/C0) of 0.01 after injection of 4 pore volumes of slurry (Fig. 3). Most of the injected nanomaterial was visibly retained at the bottom of column near the injection point. Addition of stabilizers significantly reduced the deposition of the BNM at the bottom of the column and improved the mobility in the order humic acid salt > agar agar > starch > guar gum (Fig. 3). The C/C0 values for the guar and starch stabilized slurries (in a steady state flow) reached a maximum of 0.45–0.5, compared to 0.75 for the agar agar and 0.9 for humic stabilised nanomaterials.

Indeed humic and starch coated BNM passed through the column more rapidly than the conservative tracers (KBr), and this phenomenon has been reported previously^36^. Particles, in contrast to dissolved species, can be influenced strongly by pore size exclusion effect as they move through a porous medium, and this can affect their breakthrough characteristics.The transport parameters were calculated for each of the column studies (Table 1). These include the attachment efficiency (α), particle deposition rate constant (k), single collector contact efficiency η0, predicted travel distance, 99.9% removed (LT99.9%), the predicted travel distance with 50% removed (LT50/%).

**Table 1:** Transport parameters for column studies, note the d50 of the BNM particles has been used for determination of these parameters.

**NPs**	**Effective porosity [−]**	**k [1/s]**	**η**_**0**_ **[−]**	**α [−]**	**L**_**T**_ **(50%) [m]**	**L**_**T**_ **(99.9%) [m]**
BNM uncoated	0.40	0.023	0.004	0.190	0.150	0.348
BNM in guar gum (guar is 3 g/L) in modified suspension	0.38	0.007	0.023	0.026	0.473	1.09
BNM in starch (starch is 2 g/L) in modified suspension	0.38	0.007	0.006	0.005	0.501	1.15
BNM in agar agar (agar is 2 g/L) in modified suspension	0.35	0.002	0.005	0.027	1.55	3.59
BNM in humic acid sodium salt (humic acid is 0.5 g/L) in modified suspension	0.38	0.001	0.001	0.084	2.13	4.90

### Impact of coatings on the reactivity of the bionanomagnetite

**Figure 4:**
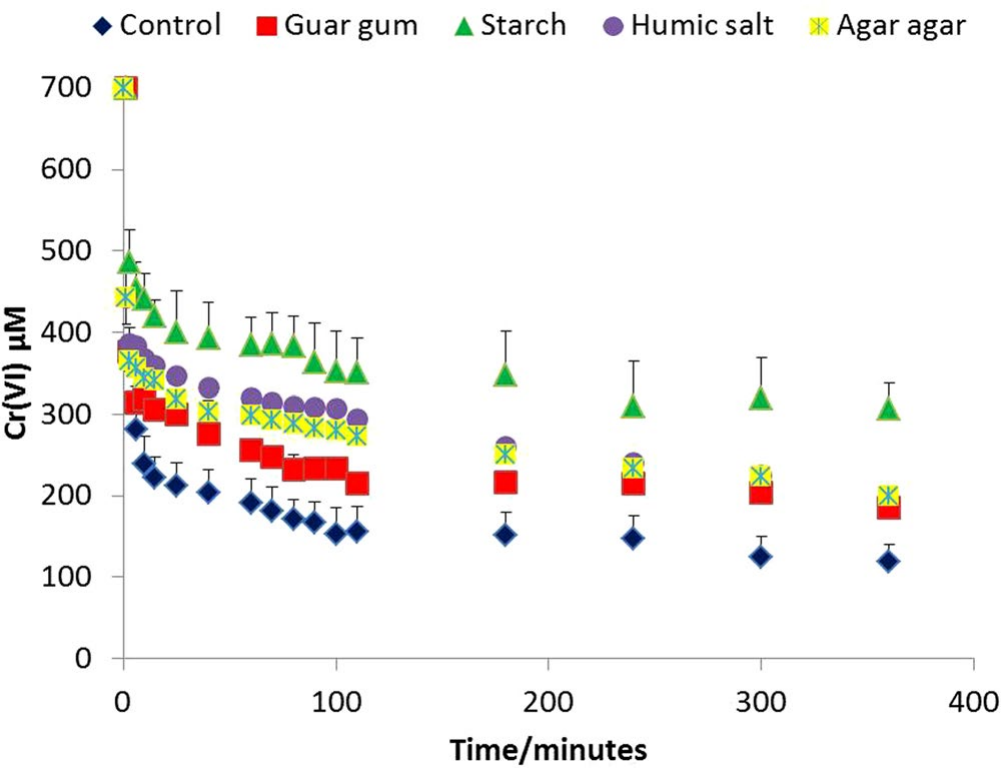
Cr(VI) concentration measured at time points are shown for coated BNM. Error bars represent standard error for triplicates of each treatment.

As the coatings tested clearly enhanced the mobility of BNM, their impact on nanomaterial reactivity was investigated by conducting batch studies using Cr(VI) as a model contaminant. BNM slurries were prepared at a concentration of 1.5 g/l total iron in synthetic soft water and Cr(VI) was added at a starting concentration of 0.7 mM, and Fe(II)-mediated reduction to Cr(III) was monitored by the diphenyl carbazide assay (DPC). The uncoated BNM showed maximum reactivity and removed 90% of the Cr(VI) within six hours. Addition of coatings inhibited Cr(VI) removal over six hours’ time, however 80% Cr(VI) was removed by guar gum coated BNM, followed by 75% removal in the case of humic-stabilized BNM (Fig. 4). Addition of a starch coating resulted in a maximal decrease in reactivity and 65% Cr(VI) removal from solution within six hours. The decrease in reactivity noted could be due to passivation of the surface of BNM with an organic coating, thereby preventing contact of Fe(II) present on the BNM surface with Cr(VI). The order of reactivity was as follows control > guar gum >humic > agar agar > starch.

### Optimization of stabilized bionanomagnetite with a Pd(0) nanocatalyst to improve reactivity

**Figure 5:**
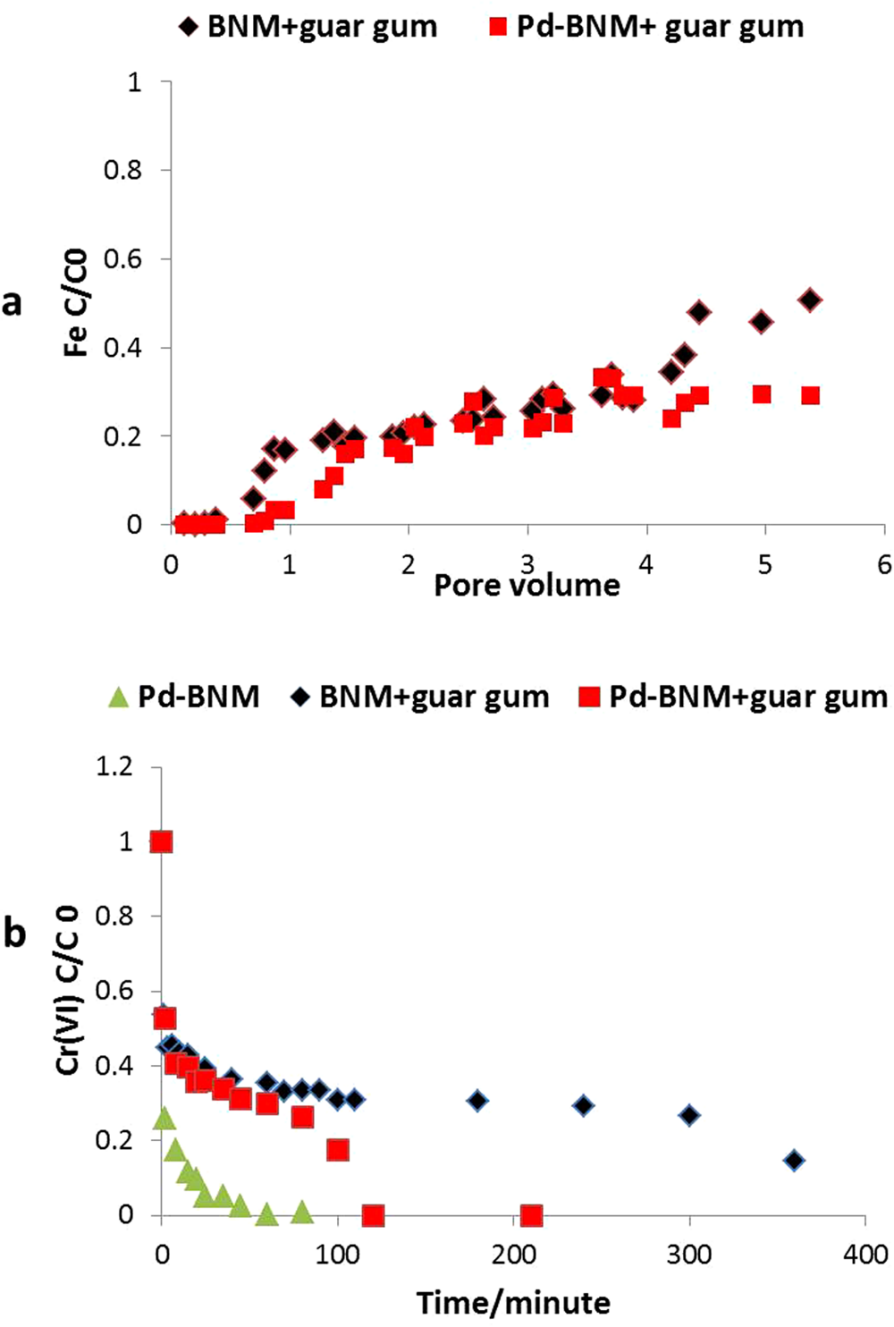
(**a**) Transport of BNM coated with guar gum (with or without Pd). The concentration of BNM at time point C, normalized to C0 (total Fe content of the slurry**)**. (**b**) Reduction of Cr(VI) by guar coated BNM, Pd-BNM with and without guar gum coatings determined by the diphenyl carbazide method.

Although the coated BNM showed improved mobility in the column studies; the presence of these coatings reduced the rate of removal of Cr(VI). In order to enhance the reactivity of the BNM, and nullify the negative impacts of coatings, the nanomaterials were functionalized with a surface array of zero valent palladium nanoparticles, as described previously^33^. Reductive precipitation of Pd(0) on the surface of BNM, after reaction with Pd(II) chloride leads to the formation of nano-scale particles decorating the surface of BNM. In the presence of an external electron donor such as hydrogen or formate, this heterocatalyst couples the sustained delivery of electrons to the prolonged and efficient reduction of Cr(VI)^13^ and other redox active contaminants^12^. Characterization of this palladized bionanomagnetite using TEM and X-ray photoelectron spectroscopy (XPS) analysis was done and details are provided in the supplementary information (Method Section S2,S4 and Figs S5,S6 respectively).This palladized biomagnetite was subsequently stabilized with guar gum or starch as model organic coatings that had been demonstrated to enhance mobility, but will be easily degraded in the environment during application, supporting the treatment of mobile plumes. Transport studies with Pd-BNM coated with these polysaccharides showed that the addition of the Pd(0) nanoparticles had a relatively minor impact on mobility,the breakthrough C/C0 was 0.35 as opposed to 0.45 when no Pd was used, Fig. 5a.

In batch reactor studies, the guar gum coated Pd-BNM removed more than 92% of the Cr(VI) added, in the presence of the electron donor H_2_ (added to the headspace) (Fig. 5b). In this reaction, less BNM was used; the total Fe equivalent loadings were 0.5 g/l compared to 1.5 g/l which was used when the non palladized BNM was tested (Fig. 4), the total Cr(VI) was also added to a final concentration of 1000 µM compared to 700 µM. These adjustments to the ratio of nanomaterial and Cr(VI) were made to account for the higher reactivity of the Pd-BNM. The guar gum coated Pd-BNM at 1/3 the iron loading showed a higher reactivity against the Cr(VI) than just the guar-coated BNM (Supplementary Table S2), mitigating against the reduction in activity associated with passivation by the organic coatings, and providing a nanomaterial with both enhanced transport properties and reactivities against redox active contaminants. A similar comparison of starch coated BNM with starch coated Pd-BNM is illustrated (Supplementary Figure S7b).

### Gamma imaging of ^99m^Tc sorbed to BNM in porous medium transport studies

**Figure 6:**
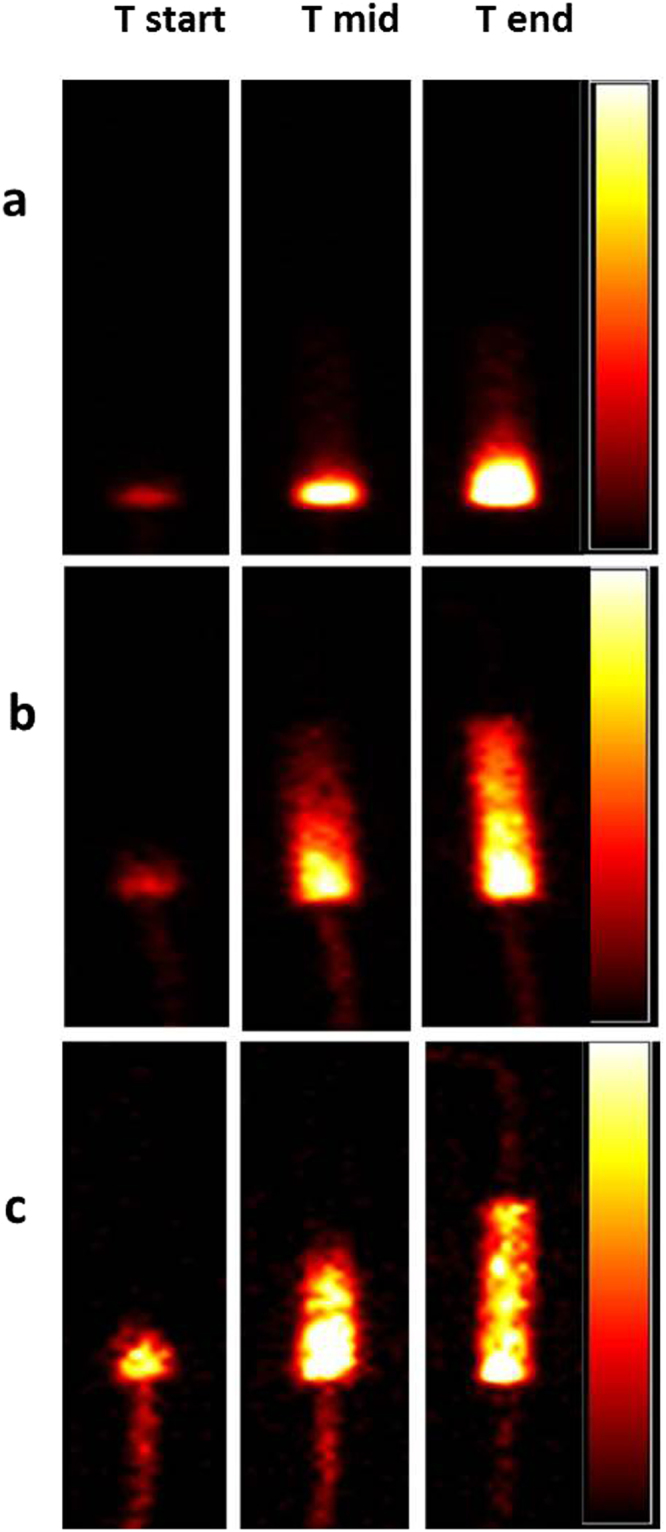
Gamma camera images of ^99m^Tc sorbed to BNM particles, illustrating the transport of the uncoated and coated BNM through columns packed with quartz sand. The top panel (a) shows uncoated BNM, the middle panel (b) guar gum coated BNM, and the bottom panel (c) humic-coated BNM. The time points shown are when the slurry first entered the column (Tstart), the mid-point of transit (T mid) and the end of experiment (T end; approximately 10 minutes).The colour scale to the right side of each panel of images represents variation in reactivity via colour change from zero activity (black) to the maximum (white) value in the image.

**Figure 7:**
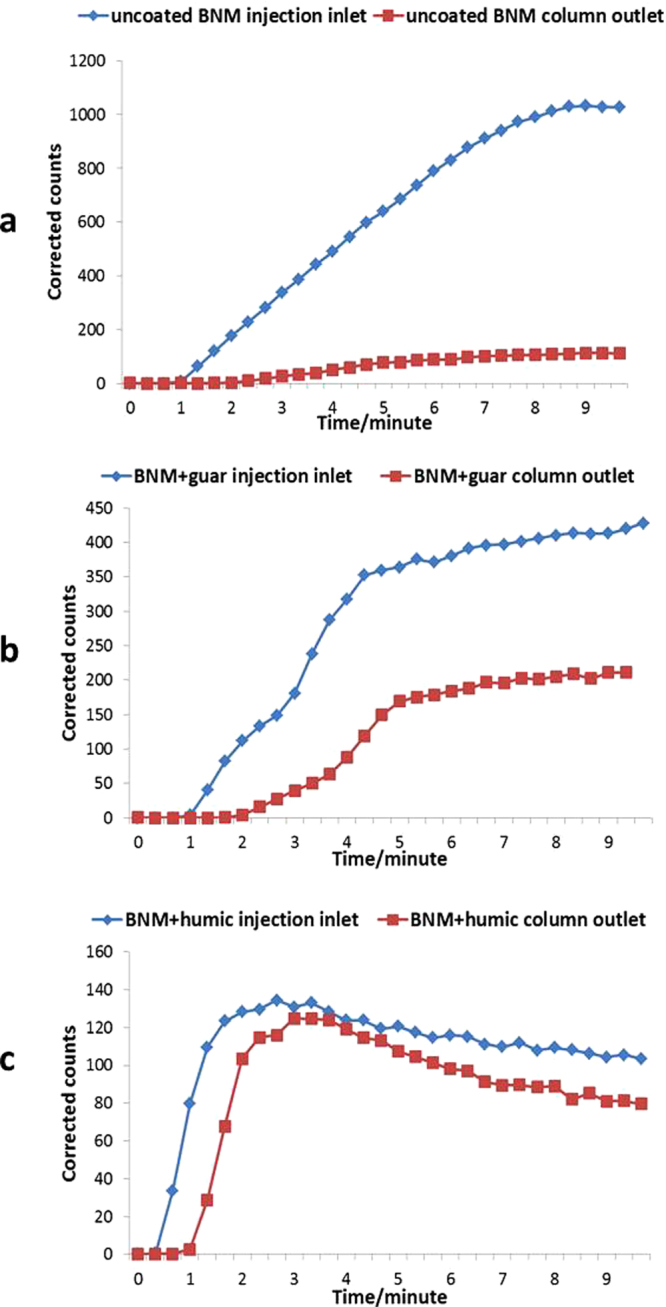
Variation of corrected counts, proportional to ^99m^Tc activity, with time in the inlet and outlet sections of columns supplied with uncoated (**a**), guar coated (**b**) and humic coated BNM (**c**).

Bionanomagnetite nanomaterial was labelled with a gamma emitting tracer that could be imaged directly during transport studies. Here a solution of ^99m^Tc(VII) (supplied as the pertechnetate anion; TcO_4_^−^) was mixed with the nanomaterial, resulting in reduction of the Tc(VII) to Tc(IV) (TcO_2_) which precipitated on the biomineral surface^37^. These suspensions were prepared in bicarbonate buffer and injected at a velocity of 100 m/d into the columns and a series of 30 gamma camera images was collected as the suspensions passed through the columns over a period of ten minutes. Figure 6 shows images of the non coated BNM control passing through the columns, compared to the guar gum-coated and humic-coated BNM. The time points shown correspond to when the suspension first entered the column, and the mid-point and end points of the experiments. Additional experiments were also conducted for 20 minutes at a lower injection velocity (50 m/d) and an Fe loading at 3 g/l (Supplementary Figure S9). Inspection of the images confirmed that the uncoated BNM moved very slowly through the column, in keeping with the breakthrough curves presented previously, and was localised close to the injection inlet of the column at the end of the imaging run (Fig. 6a, top panel). This was in stark contrast to guar gum coated and humic coated BNM suspensions, which moved through the entire length of the column (6b, middle and 6c bottom panel). Further quantitative analysis of the images for the transport studies confirmed the qualitative observations above. Figure 7a shows ^99m^Tc activity (corrected for decay) plotted against time, with the uncoated BNM showing very poor mobility; the corrected counts which reflect the concentration of ^99m^Tc are much higher in the inlet compared to the outlet, as most of labelled BNM was concentrated there (Fig. 7a). In case of guar gum and humic-coated BNM, there was a dramatic increase in ^99m^Tc levels detected at the outlet over the 10 minute experiment as the labelled BNM nanomaterial moved through the columns (Fig. 7b,c). These observations support the qualitative assessment.

## Discussion

In this study the addition of organic coatings had pronounced impacts on the physical and surface properties of aggregated BNM nanomaterial, including the size, zeta potential, settling rates and viscosity of particle suspensions. Both guar gum and starch coatings increased the size of the BNM dramatically, although the humic acid salt coating resulted in smaller (micron-scale) dimensions. Addition of an agar agar coating marginally increased the size of the BNM. However, with all treatments studied, the apparent dimensions were considerably larger than the individual crystallites (10–50 nm) as reported previously^35^, presumably due to aggregation of primary nanomaterial. We should note that these micron-scale aggregates would still considered as nanomaterials at these dimensions by the European Union as they are composed of individual particles <100 nm as noted in E.U recommendation 2011/696/EU. Similar observations have also been noted for other iron based nanoparticles synthesised by abiotic processes^38,39^, and both Vander Waal forces and magnetic forces could contribute to these phenomena^40^. In this study, the coatings were added after biosynthesis of the BNM particles from ferrihydrite. An alternative approach to stabilizing the nanoparticles could be to add the coatings before or during BNM synthesis, which is a common approach during chemical synthesis routes^39^. Initial attempts to do this with humic coatings have suggested that this approach has potential (Figure S8). Addition of the humic salt increased the net negative charge on the BNM surface,which would stabilize the nanoparticles by creating mutual repulsive forces between the particles themselves, reducing aggregation^41^. The humic coating also influenced the deposition kinetics of the BNM slurry.

Addition of positive or near neutral polysaccharides such as guar gum and starch had a lesser impact on the zeta potentials than the agar agar and humic salts. However, zetapotential is known to be a bulk parameter and represents aggregation behaviour only when significant changes occur. In fact, it has been reported to be insensitive to charge patches or heterogeneous charge distribution and cannot explain aggregation behaviour in all cases^42^. The addition of polysaccharides has been shown to increase steric repulsion and thus stabilize nanomaterial in suspension^43,44^ which could partially explain the increase in the eluting capacity of BNM (50% in the case of guar gum and approximately 80% in agar agar), which is consistent with data presented in other studies^22,38,45^. The addition of polysaccharides also increases the viscosity of the slurries, which will also enhance mobility. Finally, it should be noted that since the BNM slurry is composed of aggregates therefore some preferential filtration of larger aggregates could have occurred promoting the selective transport of smaller particles through pore throats.

The addition of coatings not only modified the physical properties of the BNM, but also affected its transport behaviour, demonstrated in a series of column studies. Of particular note is the imposition of an overall negative charge on BNM by addition of humics, which would minimise interactions with the quartz sand used in this study, and also with soils and sediments, enhancing transport during remediation applications. Here by creating a negative charge on BNM particles, less attachment to the porous medium would be expected, ensuring that fewer particles are deposited during transport.

The zeta potential is an important parameter that can control not only interactions between the particles in solution, but also interactions with geological materials. It should be noted that subsurface sediments show a high variability in terms of mineral composition, ionic strength, and natural organic material (NOM). These variables will undoubtedly influence the transport behaviour of nanomaterials, in addition to their intrinsic properties^46^. For instance, starch coated nZVI can show increased aggregation in presence of calcium ions and humic acid as these could help bridge the nanoparticles and promote aggregation^47^. Addition of all the coatings tested had significant impacts on the viscosity of the suspensions, which will in turn alter the transport properties of the BNM in the columns tested, and in soils and sediments during field injections. This was most pronounced for the agar agar and guar gum reagents. The coatings used also influenced the surface reactivity of the BNM, measured by changes in the capacity to reduce Cr(VI).This was most likely due to passivation of the Fe(II) that was accessible on the surface of the magnetite, and is known to be a potent reductant for Cr(VI), resulting in reductive precipitation of Cr(III), and incorporation into the spinel structure^11^. The reduction in the reactivity of the coated BNM could also be due to enhanced aggregation of the constituent primary particles and warrants further investigation. The rate constant for Cr(VI) reduction was highest for uncoated BNM (Table S2) and was affected significantly by the addition of the organic coatings used in this study. However, the addition of an array of Pd nanoparticles to the surface of the BNM did help recover this loss in reactivity. At an iron loading of 0.5 g/l, the Pd-BNM nanomaterial with guar gum (Fig. 5b) or starch (Supplementary Figure S7b for starch coated Pd-BNM) showed a higher reactivity in the presence of an external electron donor (Fig. 5b), and the reaction rate (K) of the BNM coated with Pd with starch or guar gum was higher than for the non palladized counterparts with just these coatings (Supplementary Table S2).

Finally, although this study has focused on Cr(VI) as a model contaminant, BNM can remediate a variety of other pollutants including organic contaminants such as PCE, TCE, nitrobenzene^14^ and azo dyes^48^. The priority radionuclides Tc(VII) and Np(V) are also amenable to treatment via BNM-mediated reduction to reduced tetravalent phases^49^. It is worth mentioning that the remediation of emerging contaminants that include pharmaceutical products (steroids), surfactants and flame retardants and pesticides, such as metaldehyde,represent future challenges that may be targeted using novel functionalized bionanomaterials^50^. The financial and environmental costs of BNM, relative to conventional synthesis routes are currently being assessed, and the use of alternative “waste” sources of iron oxides amenable to bioconversion to magnetite are a priority. Recent studies^10,15^ have shown that biosynthesis of magnetite is scalable, and revalorisation of waste or environmental sources offers a sustainable route to large-scale green chemistry production for field applications. The addition of palladium to BNM clearly enhances performance of the remediation material, although its use for environmental applications has been questioned^51^. However, as it is required in extremely low concentrations its usage may be justified for some specialised sites that are regulated and heavily contaminated, for instance, those contaminated with radioactive waste. Extraction of Pd and other metallic catalysts from waste streams may help keep the production costs low for such applications^52^, while magnetic recovery and re-use (where applicable) is feasible based on previous studies^33^.

## Conclusion

In summary we have demonstrated that the mobility of BNM can be tuned, in order to effectively utilize it in field applications for either delivery to a point source (by use of organic coatings) or usein a permeable reactive barrier (uncoated BNM). In addition the catalytic properties of the material can be optimized by adding a low loading of Pd as a nanocatalyst, extending activity for treatments including wastewater remediation^13,33^.

## Material and Methods

### Chemicals

All the reagents were procured from Sigma Aldrich unless otherwise stated.

### Synthesis of bionanomagnetite, characterization and functionalization with palladium

Bionanomagnetite was synthesized by dissimilatory reduction of ferrihydrite by the subsurface bacterium *Geobacter sulfurreducens*^53^. It was cultured in a fresh water medium^53,54^ consisting of 25 mM sodium acetate as the electron donor and 40 mM sodium fumarate as the electron acceptor at 30 °C under anaerobic conditions in the dark. The cells were harvested at late exponential phase and were added to a solution of 30 mM NaHCO_3_ supplemented with ferrihydrite (100 mM Fe, electron acceptor) and 20 mM sodium acetate (electron donor)^54^, held under an atmosphere of N2_:_CO2 (80:20 vol/vol). The method for ferrihydrite synthesis and TEM image have been provided in the supplementary information (method section S1 and Fig S2 respectively)^10,34,35^. The BNM was coated with Pd(0) as described previously^33^. It was washed in degassed water and then separated by using a bar magnet, sodium tetrachloropalladate (Na_2_PdCl_4_) solution (N_2_ purged) was then added to the BNM slurry so as to achieve a final concentration of Pd at approximately 2.4 wt%. The sample was washed twice using N_2_ purged 18.2 MΩwater under anoxic conditions and subsequently analysed for Pd loading and total Fe by ICP-MS. Further characterization was done and details have been provided in supplementary information (method section S4 and Fig. S6).

### Analytical Techniques

The mineralogical composition of BNM was determined by X-ray diffraction (XRD) using a Bruker D8 Advance diffractometer fitted with Göbel mirror in 2θ using a Cu Kα1x-ray source and diffraction data were obtained over a range of 5–70° 2θ consisting of 0.02° steps and 2 s counting times. Inductively Coupled Plasma Atomic Emission spectroscopy (ICP-AES) was performed using a Perkin Elmer Optima 5300 dual view for total iron determination after extraction in concentrated HCl (11.6 M). The zetapotential of the BNM was calculated by measuring electrophoretic mobilities (Malvern Zetasizer (Malvern Instruments, UK) and using Smoluchowski’s approximation, the volume based particle size determination (d50) was conducted using particle laser shading (time–of-transition principle, Ankersmid Eyetech^TM^ particle analyser,Nijverdal, Netherlands).

The surface area of bionanomagnetite was calculated by determining the amount of nitrogen gas adsorbed on its surface using Brunauer–Emmett–Teller (BET) theory^55^. A Gemini 2360 surface area analyser was used for this purpose. A vial containing nanoparticles was dried under Helium gas stream by using a Micromeritics Flowprep 60, and subsequently cooled under liquid N_2_. N_2_ gas was then introduced in the vial containing BNM sample and an identical empty vial, and pressure changes are recorded using a transducer in both vials. The number of molecules of nitrogen required to form a monolayer on sample surface is then calculated by the system and used to determine the surface area of sample.

### Stabilization of bionanomagnetite and sedimentation studies

The BNM was stabilized by the addition of coatings that are both non-toxic and inexpensive Moreover particles used for subsurface remediation need to be stabilized with a net negative charge, as the dominating minerals in soil and aquifers are also charged negatively (including the coating of positive charged domains with negatively charged humic matter). Hence the following coatings were used; guar gum, agar, starch and sodium salt of humic acid.These were added to a suspension of BNM (1 g/l Fe content) at different concentrations 3 g/l for the guar, 2 g/l for the agar and starch and 0.5 and 1 g/l for sodium salt of humic acid) and sonicated for 10 minutes (Grant M2 sonicator, 230 V and 50 Hz).

Preliminary screening of these slurries was conducted by measuring the sedimentation rate. The top 2.6 cm of the cylinder was marked and sampling was carefully done by aspirating a sample from the side of the cylinders without disturbing it, with a pipette and the sample was digested immediately in 0.5 M HCL in microfuge tube for Fe(II) determination using the ferrozine assay^56^. Assays were performed in triplicate. Based on these sedimentation tests, final concentrations of guar (3 g/l), agar (2 g/l), starch (2 g/l) and humic salts (0.5 and 1 g/l) were selected for column transport experiments. Here the BNM suspensions were prepared at a final concentration of 1 g/l total Fe for transport studies and 1.5 g/l total Fe for batch reactivity studies. These suspensions were prepared in a synthetic soft water of the following composition; NaHCO_3_ (48 mg/l), CaSO_4_.2H_2_O (30 mg/l), MgSO_4_ (30 mg/l) and KCl (2 mg/l). The soft water was purged with N2/CO2 (80:20) and the stabilizers added to the BNM suspensions, which were then incubated at 20 °C on shaker for 12 hours. The starting pH for all the slurries was 6.8–7.0.

### Porous medium and preparation of columns for transport studies

Dorsilit® sand was procured from Eurogrit (Netherland). It was sieved with a cut off at 0.3 mm diameter and acid washed^57^, packed into borosilicate columns (length 11.8 cm long and diameter of 3 cm), and then flushed with 10 pore volumes of soft water prior to the experiment. Norprene tubing was used throughout and the soft water delivered via a peristaltic pump (Masterflex console drive, Cole Palmer Instruments, U.K). The column assembly is illustrated in Fig. S1. The effective porosity of the medium was determined by tracer studies using KBr at 30 mg/l. An injection velocity of 100 m/d^30^ was used for delivery of the BNM, which was supplied (both coated and uncoated) at a concentration of 1 g/l equivalent total Fe. The nanoparticle slurry was purged with N_2_ gas during the experiment. The effluent from the columns was collected manually at defined time intervals (until more than 5 pore volumes of slurry was pumped through the columns). The samples exiting the column were analysed for total iron by ICP-AES. The concentration of BNM (C), at time points (t) was normalized against total concentration of iron (C0) used in the slurry, and this value was plotted against the number of pore volumes applied to the columns. All column experiments were conducted in replicates.

### Batch Cr(VI) removal experiments

Chromium(VI) reduction experiments were undertaken in acid washed bottles, each containing N_2_-purged BNM slurry (1.5 g/l total iron). It was suspended in a reactivity buffer (composition same as soft water except 2 mM NaHCO_3_ used instead). K_2_CrO_4_ solution (25 mm stock) was prepared in 18.2 MΩ water and purged with N_2_ gas. This was added to BNM stabilized by different coatings and incubated on a roller shaker in the absence of light. The samples were collected at defined intervals (time zero up to 6 hours) and a final reading was taken after 20 hours, using a N_2_ purged syringe and filtered through a filter with a 0.22 µm cut-off. Cr(VI) concentrations were determined in the filtrates by the diphenyl carbazide (DPC) assay^58^ against a standard calibration curve for known Cr(VI) concentrations and a Jenway 6715 UV/Vis spectrophotometer.For the Pd-BNM, the reaction vessel was sparged with H_2_ gas until saturation with 10 ml pure H_2_ in headspace prior to addition of Cr(VI) stock solution. The reaction rates (k_obs_) were calculated for uncoated and coated biomagnetite by the following rate equation (1).

1 $$\frac{d[Cr(VI)]}{dt}=-kobs[Cr(VI)]$$

In equation (1), [Cr(VI)] is the concentration of aqueous Cr(VI), t is time and k_obs_ is the observed pseudo-1st order rate constant. The rate was calculated by the linear regression of ln[Cr(VI)] vs time (minutes).

### Gamma imaging camera analysis and experimental set up

^99m^Tc is a metastable nuclear isomer of ^99^Tc and decays by gamma emission (short half-life of approximately 6 hours). The BNM slurry (stabilized with humic 0. 5 g/l) and guar (3 g/l) was prepared in in 20 mM sodium bicarbonate buffer at 1 and 3 g/l total Fe, and kept under anaerobic conditions. The slurries, and columns prefilled with sand saturated with soft water, were then transported to the Nuclear Medicine Centre at Central Manchester University Hospitals, where ^99m^Tc spikes of an average activity of 77 MBq and 42 MBq were applied respectively; the higher loadings of ^99*m*^ Tc were used. For the transport experiment conducted at 100 m/d, BNM (total Fe 1 g/l), theduration of the experiment was 10 minutes. For the second set, conducted at 50 m/d with Fe content 3 g/l, the duration of the experiment was 20 minutes. All slurries were incubated on a shaker at 50 RPM for 1.5 hours after the ^99m^Tc was added.

A bar magnet was used to separate the BNM from the test solution, and gamma camera imaging was then used to confirm that the ^99m^Tc activity was associated with BNM, after reduction of the soluble Tc(VII) label to insoluble Tc(IV), driven by surface localised Fe(II) on the BNM^37^. The labelled BNM was used in one set of columns at an injection velocity of 100 m/d (slurry with Fe at 1 g/l), and in a second set at 50 m/d (Fe 3 g/l). The columns were positioned vertically between two detector heads of a Siemens Symbia T6 dual headed gamma camera, fitted with low energy high-resolution collimators, so that two images were acquired simultaneously, representing the view of the columns from each side. When the ^99m^Tc-labelled BNM was injected into the columns, a series of 30 pairs of images was initiated, showing the γ-rays emitted from each column and hence the distribution of ^99m^Tc, and how this changed as the BNM was transported through the column. For the faster injection velocity, the duration of each image in the series was 20 seconds; for the slower injection velocity, it was 40 seconds.

Image analysis was conducted using GE Xeleris medical image processing software (GE Medical Systems, USA). Box-shaped regions were defined on the images from both camera heads, dividing the columns in half (inlet and outlet), with a third region over the background. The number of γ rays detected in the inlet and outlet were background-subtracted and decay-corrected to the time of the initial image in the series. The corrected counts, calculated as the geometric mean of the subtracted and corrected counts from both heads was used as an index of the radioactivity concentration in the corresponding portion of the column for each of the 30 images.

### Data availability statement

All data that weregenerated or analysed during this study are included in this published article (and its Supplementary Information file).

## Electronic supplementary material

Supplementary Information
