# Supplementary material for: Optimising the transport properties and reactivity of microbially-synthesised magnetite for in situ remediation
Source: Sci Rep. 2018 Mar 9;8:4246. doi: 10.1038/s41598-018-21733-y (PMC5844888; doi:10.1038/s41598-018-21733-y)
Supplement: Supplementary file 1 — Supplementary Information [file 41598_2018_21733_MOESM1_ESM.pdf]

Optimising the transport properties and reactivity of microbially-synthesised magnetite for *in situ* remediation

Nimisha Joshi[1], Feixue Liu [1,2], Mathew Paul Watts [1,3], Heather Williams[4], Victoria S Coker[1], Doris Schmid[5], Thilo Hofmann[5], Jonathan R Lloyd[1]

[1]School of Earth and Environmental Science, University of Manchester, Manchester M13 9PL,U.K.

[2] College of Life Sciences, University of Dundee, Dundee, DD1 5EH, Scotland, UK

[3]School of Earth Sciences, The University of Melbourne, Parkville, VIC Australia

[4] Nuclear Medicine Centre, Central Manchester University Hospitals, Oxford Road Manchester M13 9WL

[5]Department for Environmental Geosciences and Environmental Science Research Network, University of Vienna, Althnastrasse 14, 1090 Vienna, Austria

Supplementary information

S1.Ferrihydrite synthesis method

S2. TEM analysis for BNM

S3. X-ray magnetic circular dichroism (XMCD) analysis for BNM

S4.Characterization of Palladized bionanomagntite by X-ray photoelectron spectroscopy (XPS) analysis

S1. Method for synthesis of ferrihydrite

Ferrihydrite used for the bionanomagnetite was synthesized in the laboratory using alkaline hydrolysis of a $FeCl_3.6H_2O$ solution as described (Lovley and Phillips 1986), the resulting suspension was washed six times with 18.2 MΩ water to remove traces of chloride and the resulting slurry was stored at 4°C. This was analysed for iron (Fe) by ferrozine assay (Stookey 1970) and for mineral phase analysis using XRD.

Lovley, D. R. & Phillips, E. J. P. Organic matter mineralization with reduction of ferric iron in anaerobic sediments *Appl Environ Microbiol.* **52**, 51(4):683-9 (1986).

Stookey, L. L. Ferrozine-a new spectrophotometric reagent for iron. *Analytical Chemistry* **42**, 779-781 (1970).

S2. TEM analysis

The TEM imaging was conducted by using a Phillips/FEI CM200 equipped with a field emission gun, EDX system (Oxford Instruments UTW ISIS), and a Gatan imaging filter

S3. Method for XMCD analysis

Fe $L_{2,3}$-edge X-ray absorption spectra (XAS) data was collected at the Advanced Light Source (ALS), Berkeley using the octopole magnet end station (Arenholz & Prestemon, 2005) for a sample of biogenic magnetite. A sample of powder was mounted on carbon tape attached to the sample manipulator and kept in O_2-free conditions throughout. Data was collected in total-electron yield mode (probing depth of 4.5 nm) and XAS was measured for the two opposite magnetization directions by reversing the applied field of 0.6 T. The XAS spectra of the two magnetization directions were normalized to the incident beam intensity

and subtracted from each other for final XMCD spectrum. Spectra were fitted as described in Pattrick *et al.* (2002) to give the distribution of Fe(II) and Fe(III) in octahedral and tetrahedral coordination within the biogenic magnetite.

Arenholz, E. & Prestemon, S.O.Design and performance of an eight-pole resistive magnet for soft X-ray magnetic dichroism measurements. *Rev Sci Instrum.* **76(8),** 083908/1-8 (2005).

Pattrick, R.A.D., van der Laan, G., Henderson, C.M.B., Kuiper, P., Dudzik, E., and Vaughan, D.J. Cation site occupancy in spinel ferrites studied by X-ray magnetic circular dichroism: developing a method for mineralogists. *Eur J Mineral.* **14**, 1095-1102 (2002).

S4. XPS analysis of Pd-BNM

X-ray photoelectron spectroscopy (XPS) data was recorded by using a Kratos Axis Ultra employing a monochromatic Al K X-ray source and analyser pass energy of 20 eV, resulting in a total energy resolution of 0.9 eV. Uniform charge neutralization of the photo emitting surface was achieved by exposing the surface to low-energy electrons in a magnetic immersion lens system (Kratos Ltd.).The samples were dried under anaerobic conditions and loaded as powders on the spectrometer. An N_2 glove box was used to prevent exposure to oxygen. Photoelectron binding energies (BE) were referenced to C1s adventitious carbon contamination peaks set at 285 eV BE. The electron energy analyser was calibrated using elemental references: Au 4f7/2 (83.98 eV BE), Ag 3d5/2 (368.26 eV BE), and Cu 2p3/2 (932.67 eV BE). An appropriate (Shirley) background was removed from all spectra.

Figures

Figure S1a schematic figure for column transport study and S1b image of the column set up used for the experiment

Figure S2. TEM image of the lab synthesized ferrihydrite

Figure S3. XRD analysis of bionanomagnetite. Peaks assigned to magnetite are shown in red, and to siderite in blue.

Figure S 4a. XMCD image and S4b TEM image of the bionanomagnetite

Figure S5. TEM image of the palladized bionanomagnetite (Fig S5), EDX spectrum for spot analysis are shown in Fig S5(A) and S5(B) respectively.

Figure S6. XPS analysis for the Pd-BNM

Figure S7 a Cr(VI) removal comparison for palladized and guar gum coated vs just guar gum coated BNM. Fig S7b shows reactivity of palladized and starch coated BNM and starch coated BNM. The x axis shows time in minutes and y axis the Cr(VI) concentration at defined time points normalized to the starting concentration of Cr(VI) in µM. The error bars reprsent standard error for triplicates used for each of the treatments.

Figure S8: Zeta potential values for humic stabilized BNM, where humic salt was added during ferrihydrite synthesis (precursor stage). Error bars represent the standard error for triplicate measurements of the sample.

Figure S9: Gamma camera images of ^{99m}Tc sorbed to BNM particles (3 g/l total Fe) at 50 m/d, illustrating the transport of the uncoated and coated BNM through columns packed with quartz sand. The left panel (a) shows uncoated BNM, the middle panel (b) guar-coated BNM, and the right panel (c) humic-coated BNM. The time points shown are when the slurry first

entered the column (T start), the mid-point of transit (T mid is 10 minutes) and the end of experiment (T end is 20 minutes).

Tables

Table S1 size characterization of BNM with and without coatings

Table S2 Reaction rate k $_{obs}$ (per minute, per hour and normalized to total iron loading) have been provided for uncoated, coated BNM and also for palladized and stabilized BNM. The values in brackets represent the BNM concentration as represented by total iron loading in the reaction vessel.

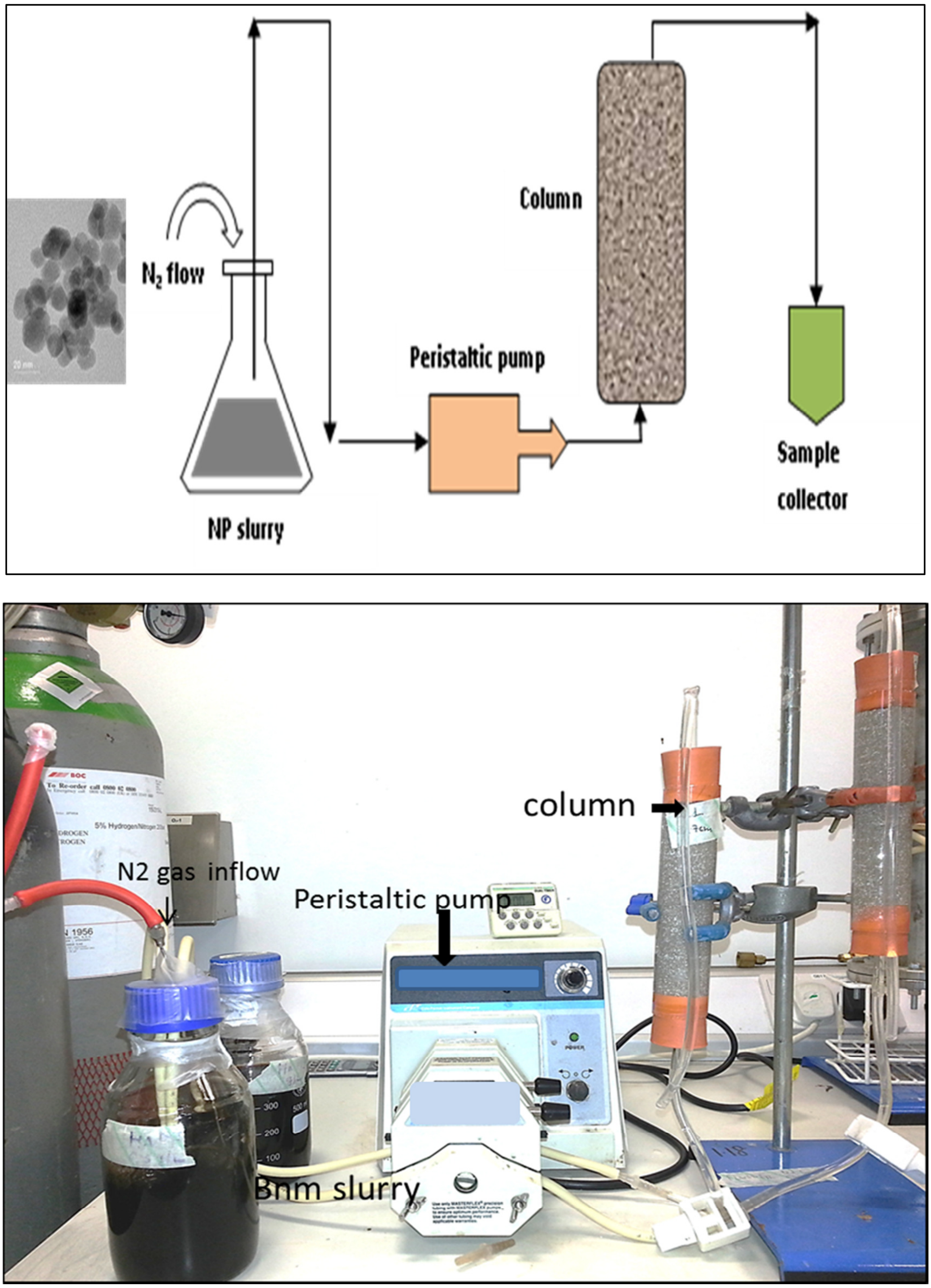
a
Column
N_2 flow
Peristaltic pump
Sample
collector
NP slurry
b
column
N2 gas inflow
Peristaltic pump
Bnm slurry

Figure S1

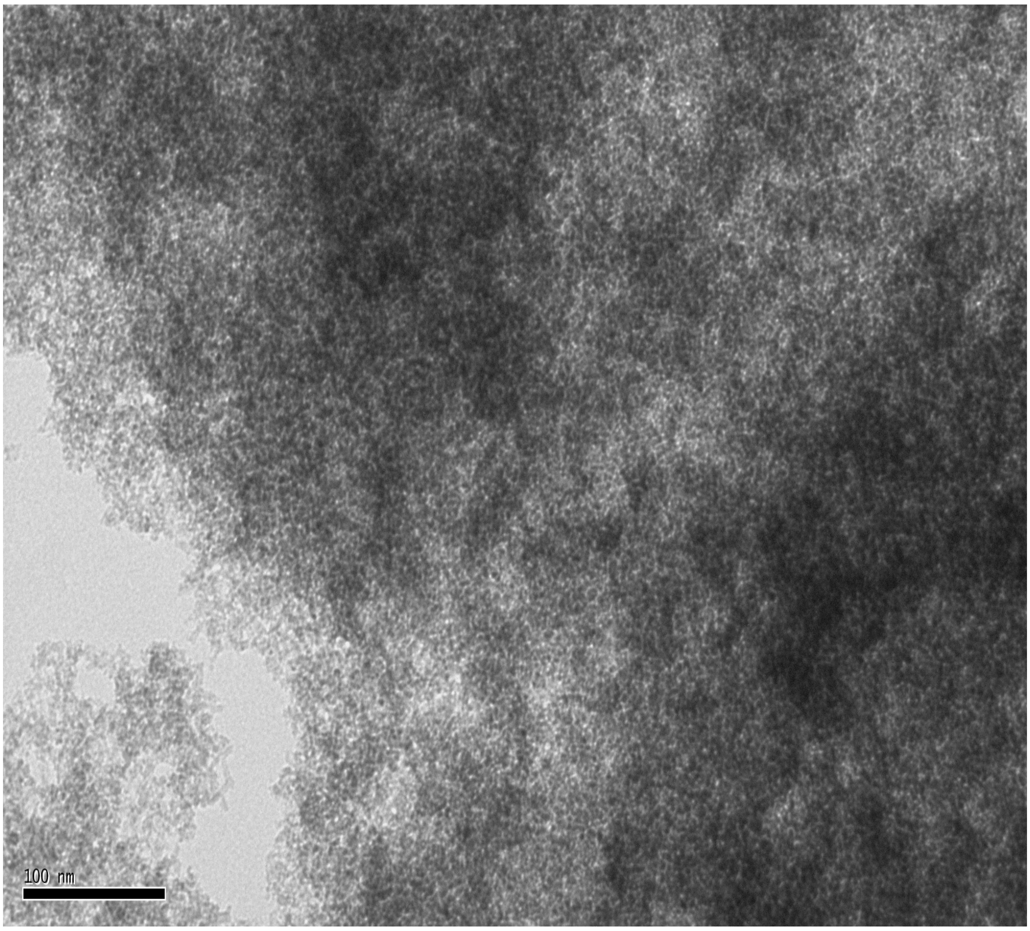

Figure S2

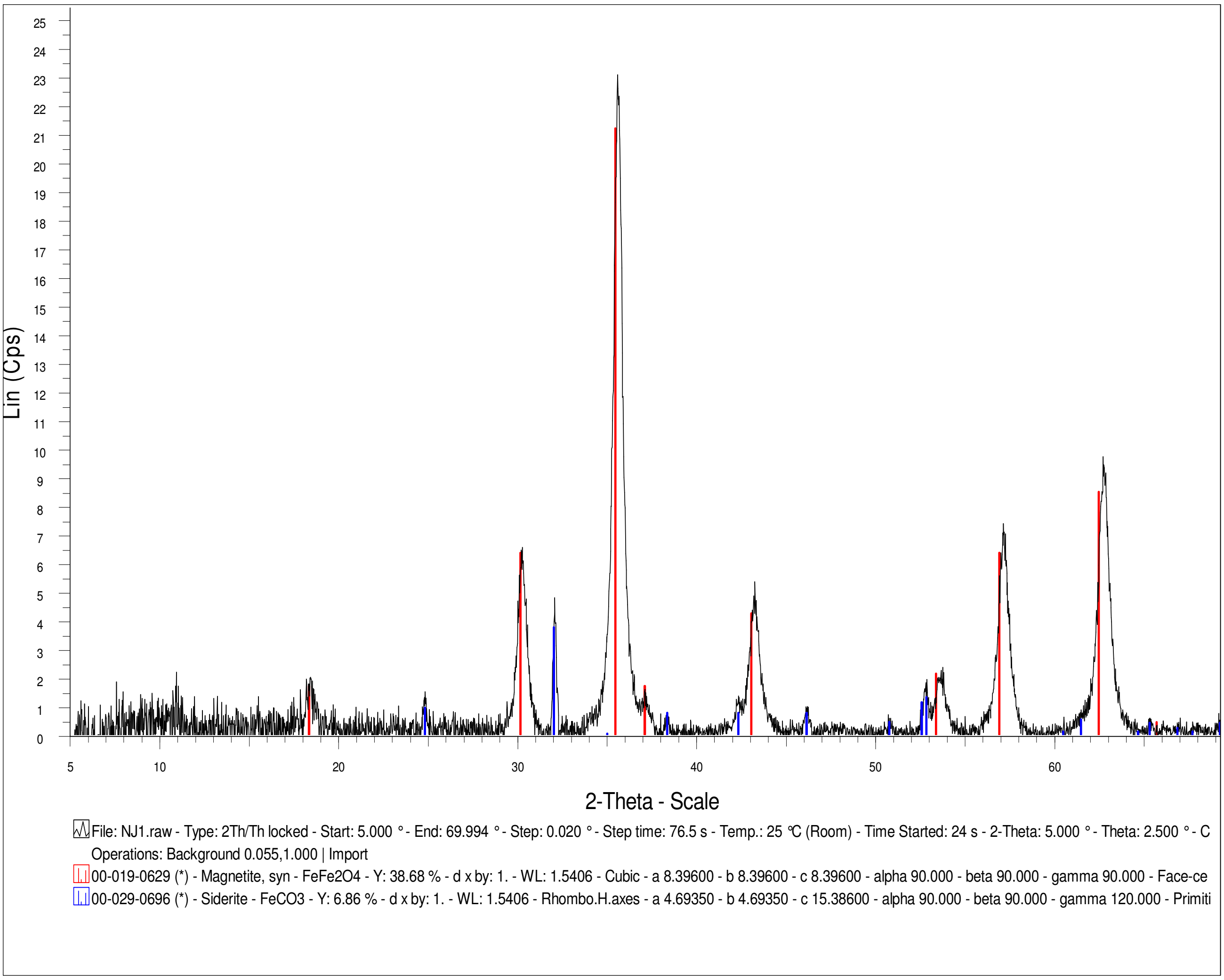
Lin (Cps)
2-Theta - Scale
File: NJ1.raw - Type: 2Th/Th locked - Start: 5.000 ° - End: 69.994 ° - Step: 0.020 ° - Step time: 76.5 s - Temp.: 25 °C (Room) - Time Started: 24 s - 2-Theta: 5.000 ° - Theta: 2.500 ° - C
Operations: Background 0.055,1.000 | Import
00-019-0629 (*) - Magnetite, syn - FeFe2O4 - Y: 38.68 % - d x by: 1. - WL: 1.5406 - Cubic - a 8.39600 - b 8.39600 - c 8.39600 - alpha 90.000 - beta 90.000 - gamma 90.000 - Face-ce
00-029-0696 (*) - Siderite - FeCO3 - Y: 6.86 % - d x by: 1. - WL: 1.5406 - Rhombo.H.axes - a 4.69350 - b 4.69350 - c 15.38600 - alpha 90.000 - beta 90.000 - gamma 120.000 - Primiti

Figure S3

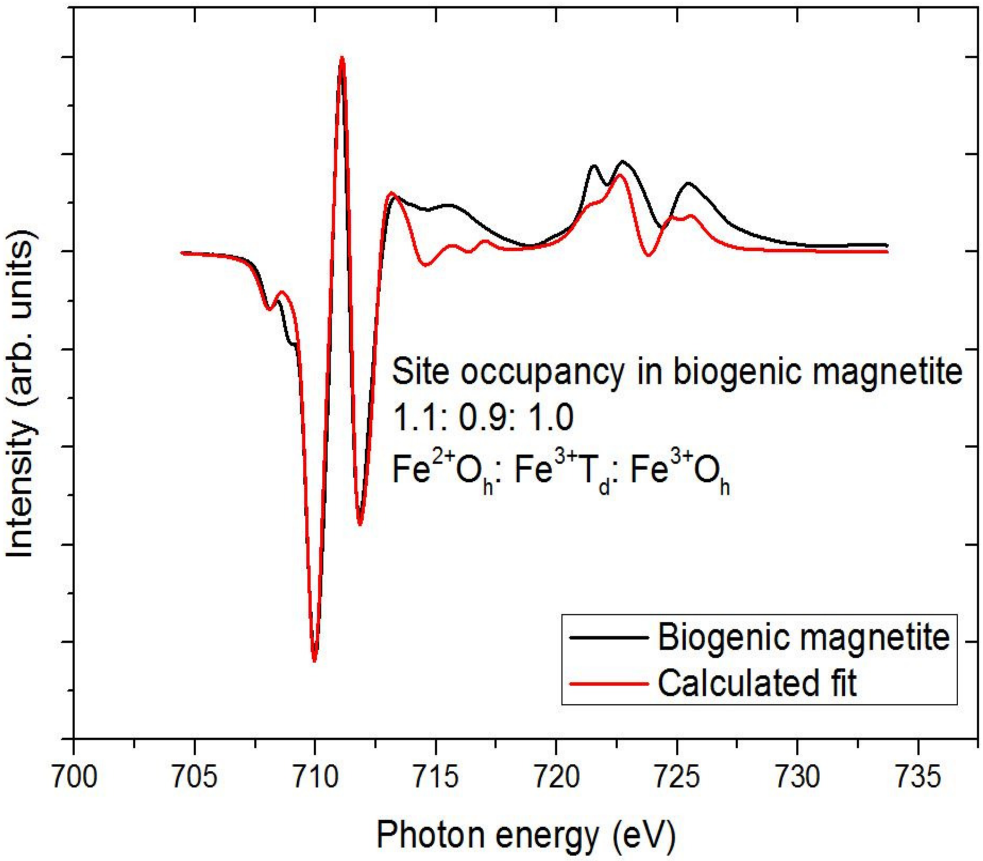

Figure S4a

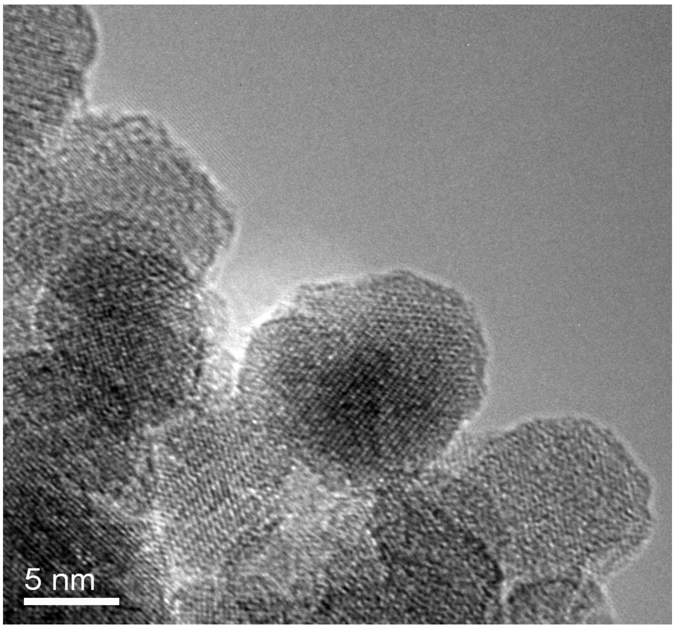

Figure S4b

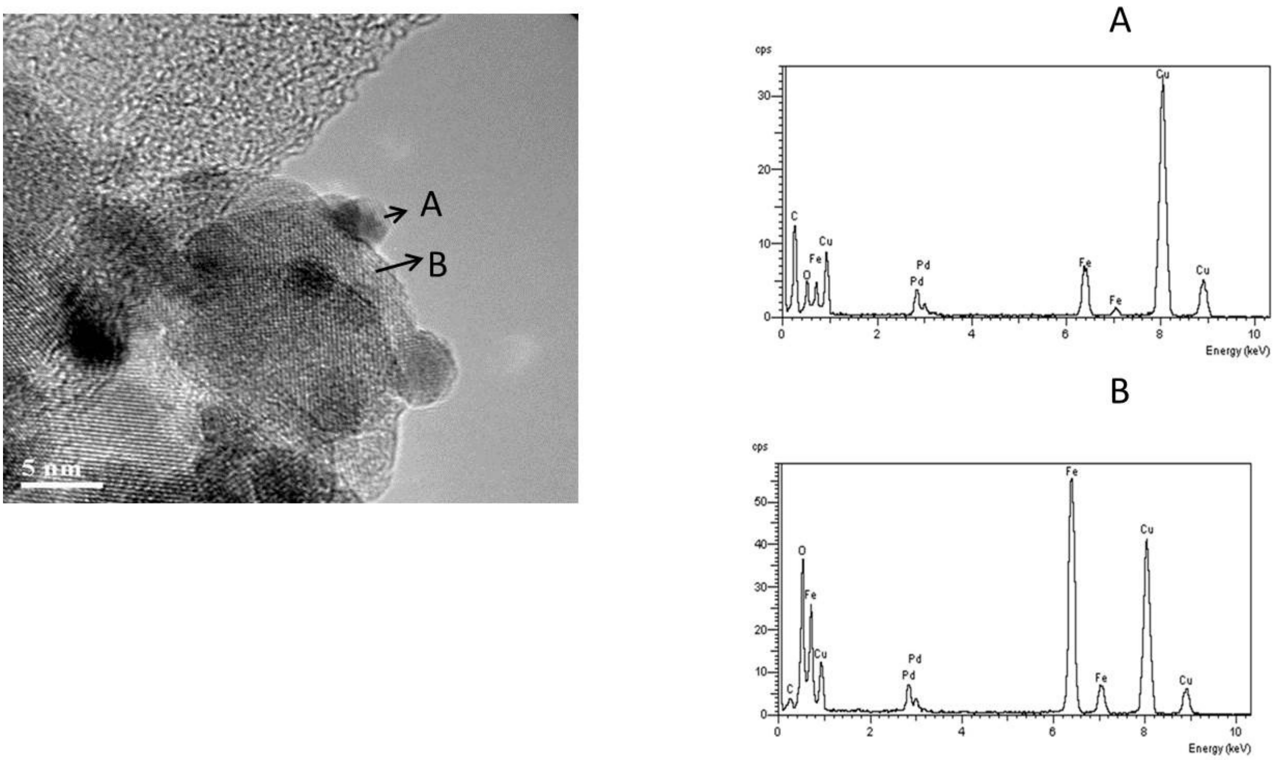

Figure S5

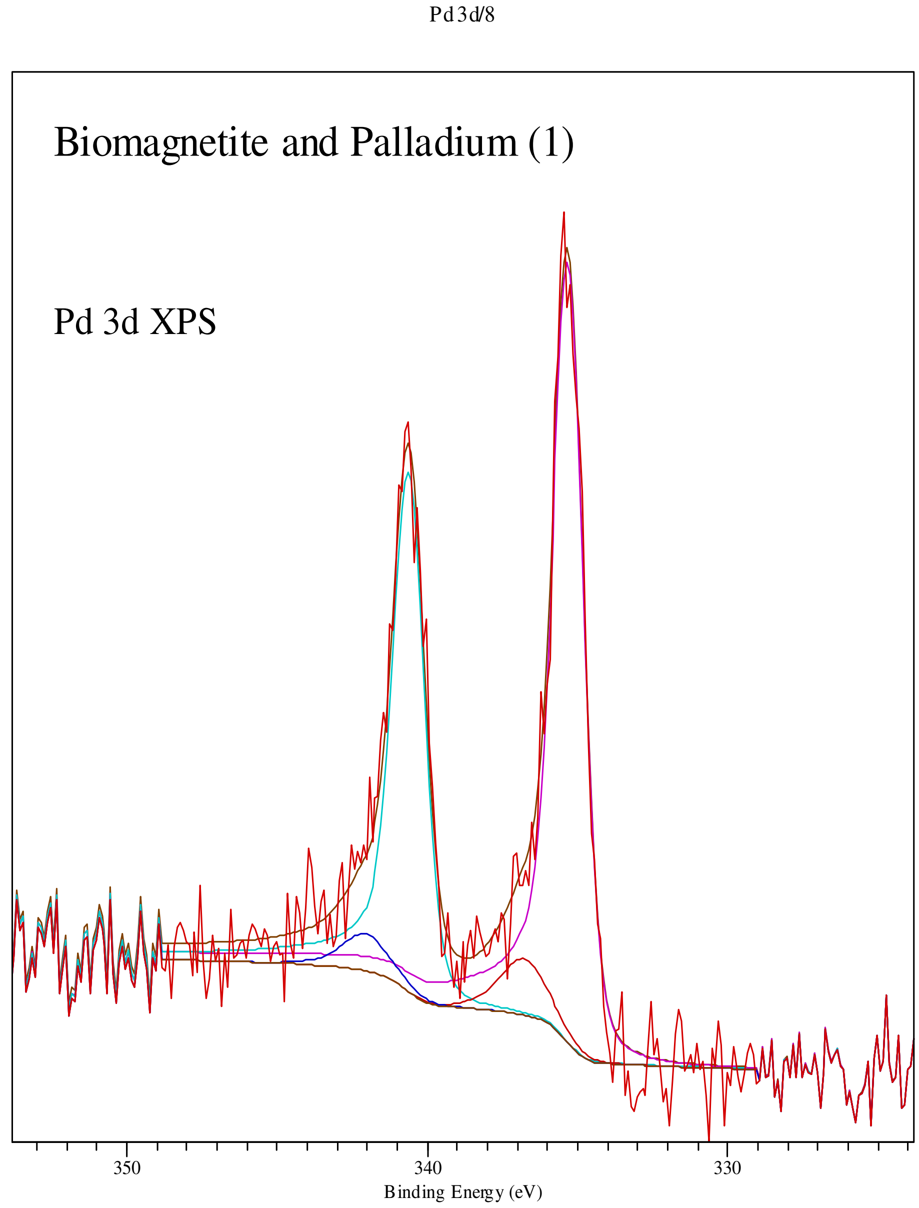
Pd 3d/8
Biomagnetite and Palladium (1)
Pd 3d XPS
350
340
330
Binding Energy (eV)

Figure S6

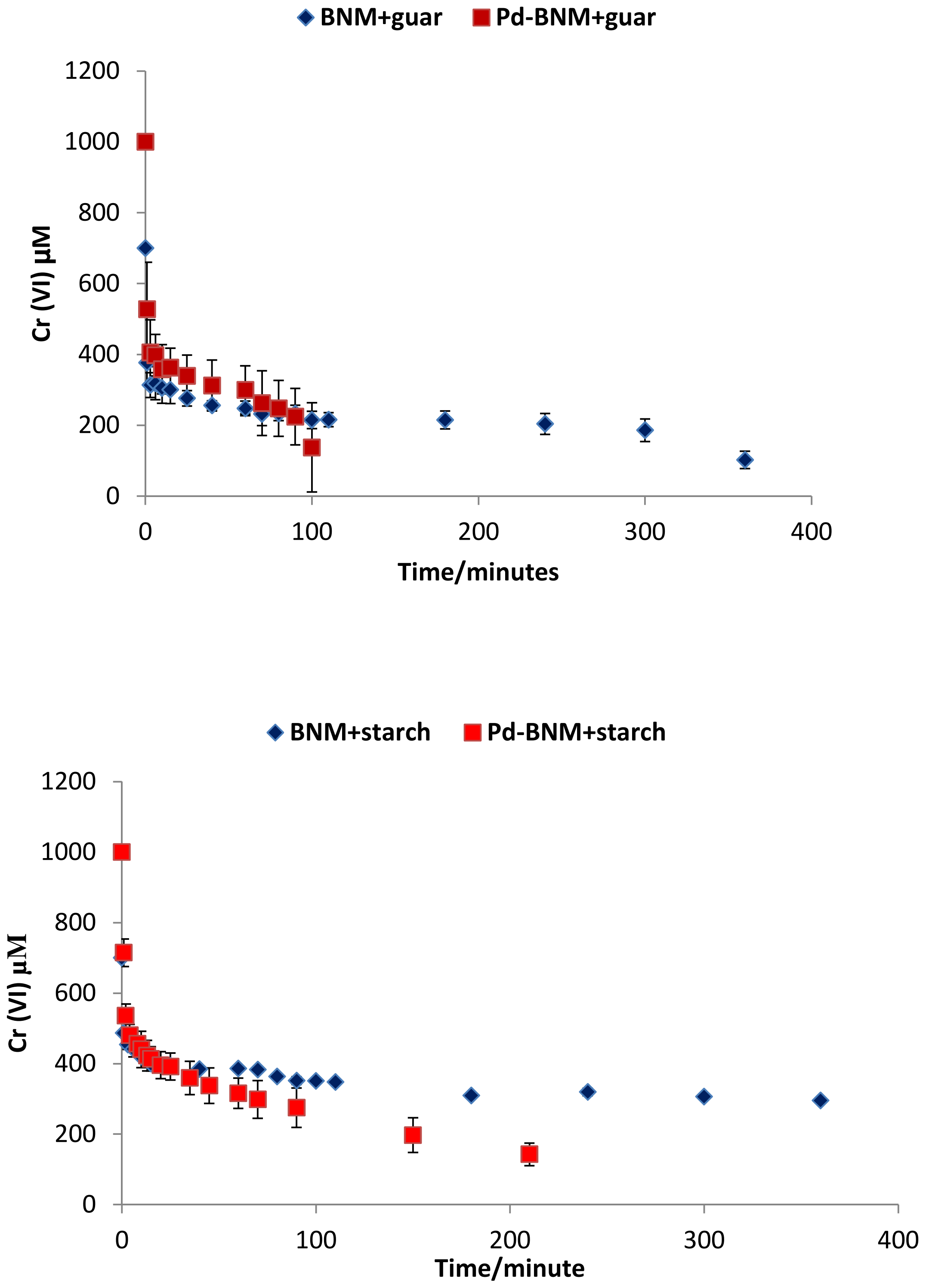

Figure S7a (top) and S7b (bottom)

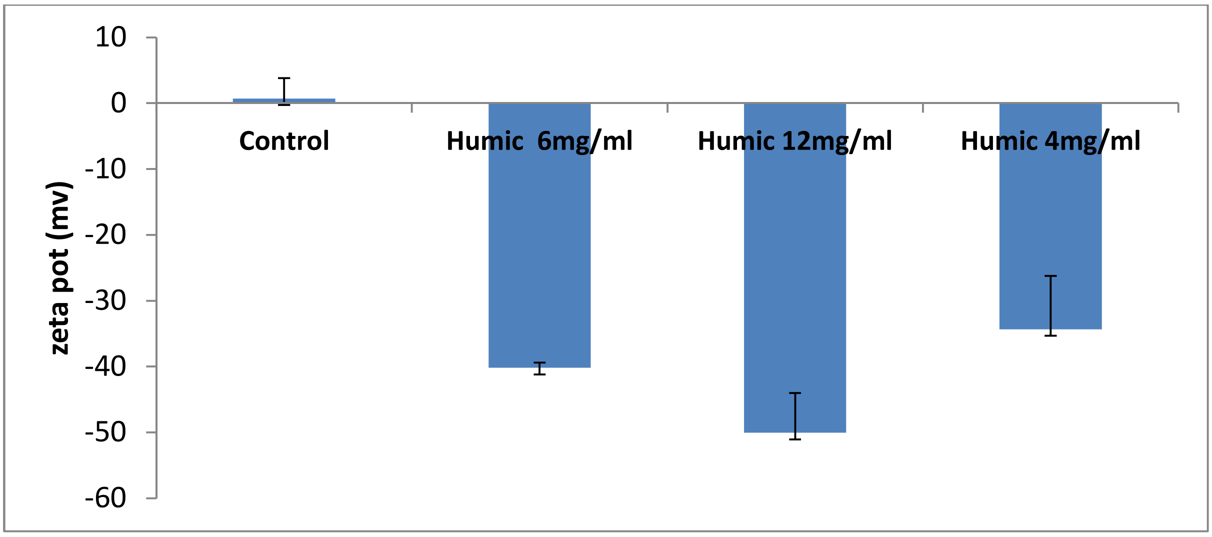
10
0
-10
-20
-30
-40
-50
-60
zeta pot (mv)
Control
Humic 6mg/ml
Humic 12mg/ml
Humic 4mg/ml

Figure S8

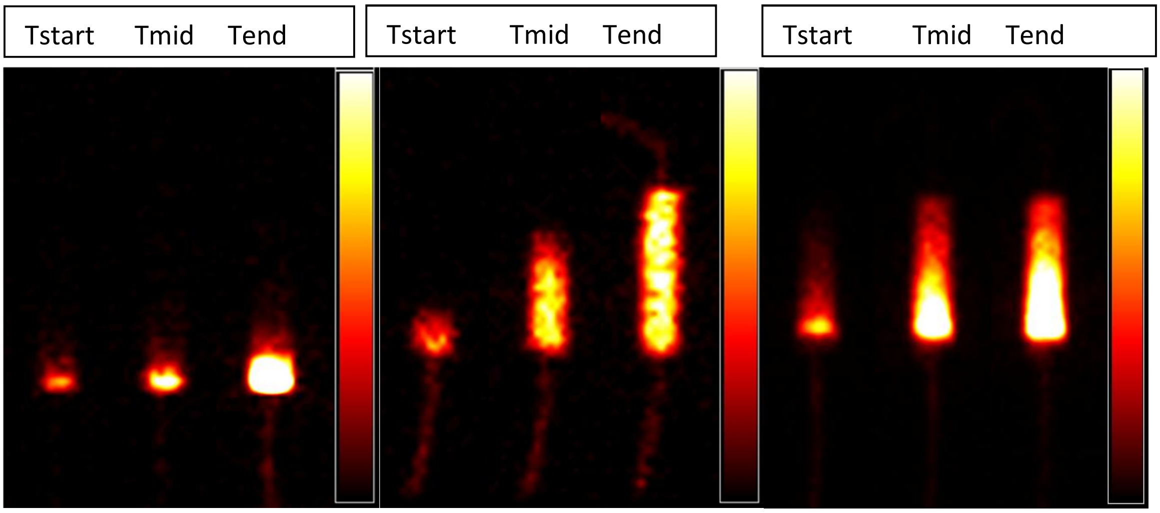

Figure S9a **S9b** **S9c**

Figure S9

**Bionanomagnetite in different suspensions**	**Average particle diameter [µm]**	**d_{10} [µm]**	**d_{50} [µm]**	**d_{90} [µm]**
BNM in soft water matric	3.50	3.90	5.70	7.70
BNM + guar gum suspension	13.13	5.96	12.30	21.49
BNM + agar agar suspension	5.52	2.82	5.49	8.31
BNM +starch suspension	23.49	7.84	22.43	41.25
BNM +sodium humate suspension (0.5 g/L)	3.22	1.41	2.67	5.65

Table S1

**Samples**	**R^2**	**Rate (k $_{obs}$/min)**	**Rate per hour (kh^{-1})**	**$kh^{-1}g^{-1}$ (rate per hour normalized to mass loading of iron (g/l) in batch reactor)**
Uncoated BNM (control) (Fe 1.5 g/l)	0.977	0.0482	2.892	1.928
Guar stabilized (Fe 1.5 g/l)	0.946	0.045	2.7	1.8
Starch stabilized (Fe 1.5 g/l)	0.9277	0.004	0.24	0.16
Agar agar (Fe 1.5 g/l)	0.965	0.005	0.3	0.2
Humic coated BNM (Fe 1.5 g/l)	0.965	0.0053	0.32	0.212
Pd -BNM control (Fe 0.5 g/l)	0.959	0.369	22.14	44.28
Pd -BNM guar (Fe 0.5 g/l)	0.935	0.0507	3.042	6.084
Pd- BNM starch (Fe 0.5 g/l)	0.9611	0.0555	3.33	6.66

Table S2
